# Food Components and the Immune System: From Tonic Agents to Allergens

**DOI:** 10.3389/fimmu.2013.00102

**Published:** 2013-05-17

**Authors:** Ana Maria Caetano Faria, Ana Cristina Gomes-Santos, Juliana Lauar Gonçalves, Thais Garcias Moreira, Samara Rabelo Medeiros, Luana Pereira Antunes Dourado, Denise Carmona Cara

**Affiliations:** ^1^Departamento de Bioquímica e Imunologia, Instituto de Ciências Biológicas, Universidade Federal de Minas GeraisBelo Horizonte, Minas Gerais, Brazil; ^2^Instituto de Investigação em Imunologia (iii)São Paulo, Brazil; ^3^Laboratório de Interação Microorganismo-Hospedeiro, Departamento de Microbiologia, Universidade Federal de Minas GeraisBelo Horizonte, Minas Gerais, Brazil; ^4^Laboratório de Imunofarmacologia, Departamento de Bioquímica e Imunologia, Universidade Federal de Minas GeraisBelo Horizonte, Minas Gerais, Brazil; ^5^Departamento de Morfologia, Instituto de Ciências Biológicas, Universidade Federal de Minas GeraisBelo Horizonte, Minas Gerais, Brazil

**Keywords:** nutrition, oral tolerance, food allergy, diet, CLA, vitamin A, food aversion

## Abstract

The intestinal mucosa is the major site of contact with antigens, and it houses the largest lymphoid tissue in the body. In physiological conditions, microbiota and dietary antigens are the natural sources of stimulation for the gut-associated lymphoid tissues (GALT) and for the immune system as a whole. Germ-free models have provided some insights on the immunological role of gut antigens. However, most of the GALT is not located in the large intestine, where gut microbiota is prominent. It is concentrated in the small intestine where protein absorption takes place. In this review, we will address the involvement of food components in the development and the function of the immune system. Studies in mice have already shown that dietary proteins are critical elements for the developmental shift of the immature neonatal immune profile into a fully developed immune system. The immunological effects of other food components (such as vitamins and lipids) will also be addressed. Most of the cells in the GALT are activated and local pro-inflammatory mediators are abundant. Regulatory elements are known to provide a delicate yet robust balance that maintains gut homeostasis. Usually antigenic contact in the gut induces two major immune responses, oral tolerance and production of secretory IgA. However, under pathological conditions mucosal homeostasis is disturbed resulting in inflammatory reactions such as food hypersensitivity. Food allergy development depends on many factors such as genetic predisposition, biochemical features of allergens, and a growing array of environmental elements. Neuroimmune interactions are also implicated in food allergy and they are examples of the high complexity of the phenomenon. Recent findings on the gut circuits triggered by food components will be reviewed to show that, far beyond their role as nutrients, they are critical players in the operation of the immune system in health and disease.

## Introduction

The intestine is the largest interface between the body and the external environment (Mayer, 2005). Most contacts with foreign antigenic materials occur at the gut mucosa. It has been reported that 130–190 g of protein is absorbed in the small intestine daily (Brandtzaeg, 1998) and the gastrointestinal tract harbors approximately 10^14^ microorganisms of more than 1000 species (Gill et al., 2006). Moreover, it is also known that the intestine houses the most abundant lymphoid tissue in the body. There are 10^12^ lymphoid cells per meter of human small intestine. In mice and humans, the number of immunoglobulin (Ig)-secreting cells located in the gut exceeds by several fold the number found in other lymphoid organs altogether (Mestecky and McGhee, 1987; van der Heijden et al., 1987). Lymphocytes that compose the gut-associated lymphoid tissue (GALT) are either part of lymph-node-like structures such as Peyer’s patches (PP) or scattered throughout the lamina propria and intraepithelial spaces of the intestine in such a way that it is impossible to dissociate functionally epithelia and lymphoid components. B-cell-deficient mice, for instance, have a defect in the formation of the specialized epithelial cell called M cell (Alpan et al., 2001). More recently, it has been shown that a unique population of CCR6(hi)CD11c(int) B cells resides in the subepithelial dome (SED) in mouse PP and that they are essential for M cell maturation (Ebisawa et al., 2011). Thus, B lymphocytes are not only lodged in the intestine but they also provide signaling molecules for the differentiation of the gut epithelia. This specialized surface is covered by a single layer of epithelial cells with absorptive properties suggesting that the gut mucosa represents an interface, rather than a barrier, between the inside (self) and the outside (non-self). Materials coming from the environment find in the enterocytes and M cells a selective but permeable gate of entrance into the body. It is expected that contact with these antigenic materials has an important impact in the general activity of the immune system (Faria and Weiner, 2005).

Currently, several researches are involved in the effort to understand the role and function of microbiota in the activity of the immune system (Lee and Mazmanian, 2010; Maslowski and Mackay, 2011). However, the stimulation provided by food antigens has been addressed only by a handful of studies. Most of them examined the indirect effect of many food components in immunity through the modulation of gut microbiota. This review presents a critical view of the direct influence of dietary components in the physiological operation of the immune system, in the maintenance of intestinal homeostasis, as well as in the development of adverse reactions to food proteins.

## Food Proteins in the Maturation of Immune System

Feeding is the major and most frequent occasion in which the organism contacts foreign proteins and other immunologically relevant molecules (Faria and Weiner, 2005). Nutrients have biological and immunological importance in all stages of life. They are essential materials for the survival of organisms having a great impact in the body’s physiology from their absorption, metabolism until their excretion.

Metchnikoff, in his pioneer work on the role of phagocytosis in immunity, had already pointed out that even at a cellular level, eating and digestion are fundamental activities by which immune cells perform their tasks (Tan and Dee, 2009). A collection of recent findings on antigen presentation as well as on the signaling pathways triggered by amino acids, lipids, and vitamins confirm the critical role of dietary components and nutrition in the basic function of the immune system.

The first contact of the immune system with nutrients occurs in embryogenesis. Nutrients from the maternal circulation are the structural basis for the development of new body tissues. The diversity, quality, and quantity of these nutrients modulate the functional characteristics of body tissues. After birth, breastfeeding also provides immune cells, immunoglobulins, and important cytokines, such as TGF-β, that are key players in the immunological setting of the newborn (Kainonen et al., 2012).

The relationship between nutrition and immunity was first observed in clinical investigations of malnutrition and in animal models with nutritional deficiencies. Malnutrition is defined as “the imbalance between supply of nutrients and energy and the body’s demand for them to ensure growth, maintenance, and specific functions.” Protein energy malnutrition (PEM) increases susceptibility to respiratory and gastrointestinal infections and the severity of many infectious diseases. Malnutrition can be directly associated with several defects in the functioning of the immune system (as summarized in Table 1).

**Table 1:** **Immunological changes in malnutrition**.

	Human	Animal
Thymus	Reduction in cortical thymocytes (Lyra et al., 1993)	Apoptosis of immature CD4^+^CD8^+^ lymphocytes
		Decrease thymocyte proliferation (Chandra, 1992)
Spleen	–	Reduction in the number of mononuclear cells (Manhart et al., 2000)
Bone marrow	Increased proportion of neutrophil granulocytes (Wickramasinghe et al., 1988)	Decrease of hematopoieitic stem cells (Xavier et al., 2007)
Peyer’s patches	–	Increase in B cells
		Reduction in the number of mononuclear cells (Manhart et al., 2000)
Secretory IgA	Enhanced synthesis [Secretory IgA synthesis in Kwashiorkor (Beatty et al., 1983)]	Reduction of luminal content (Manhart et al., 2000)
	Significant decrease in sIgA-containing cells in the jejunal mucosa (Green and Heyworth, 1980)	
Gut microbiota	Children with kwashiorkor significant decrease in Actinbacteria (Smith et al., 2013)	The relative proportion of *Bilophila wadsworthia* were higher in C57BL/6 mice with the children’s kwashiorkor microbiota (Smith et al., 2013)


Since the 70’s, clinical and epidemiological studies showing a correlation between malnutrition and infectious diseases substantially increased. A high prevalence worldwide of malnutrition related enteric diseases is especially important in neonates (CDC, 2008).

Morphological atrophy of lymphoid tissues is a common feature in PEM. Thymus atrophy, known as “nutritional thymectomy” is the most well described alteration in lymphoid tissues in the context of malnutrition (Chandra, 1992; Savino et al., 2007); (Prentice, 1999). Malnutrition increases apoptosis of immature double positive CD4^+^CD8^+^ lymphocytes and decreases thymocyte proliferation (Chandra, 1992) as demonstrated in rodents by the reduction of proliferating cell nuclear antigen (PCNA) expression in thymocytes (Mitsumori et al., 1996). In humans, there is a significant reduction in cortical thymocytes that could be detected in autopsy of malnourished patients (Lyra et al., 1993). Importantly, restoration of nutritional status reverses thymic atrophy (Chevalier et al., 1996). Bone marrow also suffers with malnutrition. Malnourished mice have severe bone marrow atrophy confirmed by a reduction of PCNA. These animals have more fibronectin accretion in paratrabecular and endosteal regions and more laminin deposition in perisinusal sites. In addition, there is a decrease in the ability of hematopoietic stroma of malnourished mice to support the growth of hematopoietic stem cells (CD34^+^) *in vitro* (Xavier et al., 2007).

Protein malnutrition (PM) has an impact on IgA production and on the number and phenotype of lymphocytes in PP and spleen. Mice fed a protein-deficient diet for 4 days show a significant reduction in the number of mononuclear cells in these organs. There was a relative increase of B cells in the PP, the luminal IgA content of small intestine was significantly diminished after 4 days of PM and remained reduced until 10 days of PM. Expression of the costimulatory molecules CD80 and CD86 on B cells was upregulated in PP but markedly downregulated in the spleen, which was inversely related to the expression of the counter receptor CD28 on helper T cells (Manhart et al., 2000).

There is also evidence of damage in the intestinal mucosa during malnutrition. In an animal model of septicemia induced by zymosan, protein malnourished mice had bacteria translocation from the gut to the liver, spleen, and blood stream. Zymosan-induced bacterial translocation appeared to be related to the combination of mucosal injury and a disruption in microbiota composition of malnourished mice (Deitch et al., 1990). The relationship between malnutrition and microbiota has been explored recently and represents a promising field of research to define mechanisms and treatment of malnutrition. Smith et al. (2013) findings implicate the gut microbiome as a causal factor in kwashiorkor, a severe acute form of malnutrition. They studied 317 Malawian twin pairs during the first 3 years of life. Children with kwashiorkor manifested a statistically significant decrease in Actinobacteria with the introduction of RUTF (ready to use therapeutic food) unlike their healthy co-twins. The transplanting of fecal microbial communities, obtained from kwashiorkor children, into gnotobiotic mice, combined with a typical diet of Malawi, resulted in significantly greater weight loss in recipient mice when compared to animals that received the healthy sibling’s microbiota. The relative proportion of *Bilophila wadsworthia*, a sulfite-reducing, immunogenic microbe detected under pathological conditions such as appendicitis and intestinal inflammatory disorders, was higher in mice with the kwashiorkor microbiota. Previously, Devkota et al. (2012) showed that *B. wadsworthia* expansion induced by taurocholic acid after a milk-fat-enriched diet was associated with Th1 responses and increased incidence of colitis in interleukin (IL)-10^−/−^ mice. Hashimoto and coworkers also studied the mechanisms by which unbalanced dietary nutrients affect microbial ecology and intestinal homeostasis. They reported that deficiency in angiotensin I converting enzyme (peptidyl-dipeptidase A) 2 causes a critical disturbance in the intestinal tryptophan homeostasis that alters the susceptibility to gut inflammation (Hashimoto et al., 2012). These results show the existence of a microbial profile correlated with the development of malnutrition secondary to inflammatory damage to the intestinal epithelial cells.

These reports clearly point to the role of an appropriate supply of dietary proteins in the formation and maintenance of lymphoid structures such as the gut mucosa. However, we believe that these molecules may play roles beyond the ones typically understood as nutritional functions. There is strong evidence that nutrients are required for the early establishment and maintenance of gut function, even when there is not a context of malnutrition. Presence of intact proteins in the diet has a critical role in the development and maturation of the immune system. Although most dietary macromolecules are degraded by the time they reach the small intestine, both in humans and rodents, some undegraded or partially degraded proteins are absorbed into the blood in an immunogenic form (Husby et al., 1985; Bruce et al., 1987). To address the effect of food protein stimulation on the development of immune system, our research group developed an isocaloric balanced diet in which dietary proteins were replaced by equivalent amounts of amino acids (Menezes et al., 2003). Because amino acids are very small, they are not presented in major histocompatibility complex (MHC) groove and are not considered antigenic molecules. All other nutrients were kept in their intact form. To test the effect of feeding this special diet in the maturation of the immune system, C57BL/6 mice were reared from weaning to adulthood on this whole-protein-deprived diet (Aa diet) in open cage conventional conditions. Adult Aa-fed mice (12 weeks of age) showed no sign of malnutrition, but they presented several local and systemic immunological alterations. They had poorly developed GALT with smaller Peyer’s patches, reduced number of intraepithelial lymphocytes (IELs) and lamina propria cells. Production of secretory IgA was drastically reduced. Serum levels of IgG and IgA, but not IgM, were also significantly lower, and the cytokine profile produced by T cells from several lymph nodes revealed a systemic skewing toward a Th2 profile. Thus adult Aa-fed mice resemble sucking mice suggesting that antigenic stimulation by food proteins after weaning plays an essential role in the maturation of immune system. Adult mice fed an elemental diet since weaning present similar defects in immune function (Menezes et al., 2006).

As expected, when adult Aa-fed C57BL/6 mice were challenged with *Leishmania major* infection, they show an increased susceptibility to infection, slower clearance of the parasite along with low levels of IL-12, IFN-γ, and nitric oxide production. Experimental infection with *L. major* has established the Th1/Th2 paradigm. While infected C57BL/6 mice develop a Th1 response that results in IFN-γ production, macrophage activation, and control of parasite growth, BALB/c mice respond to this parasite by developing a Th2 response that is associated with inefficient macrophage activation and compromised control of parasite growth (Heinzel et al., 1989; Alexander and Bryson, 2005). We observed that the impaired Th1 polarization in C57BL/6 Aa-fed mice is related to an immature state of antigen-presenting cells (APCs) in Aa-fed mice rather than an intrinsic defect in T cells. (Amaral et al., 2010). Phenotypical and functional analysis of APCs from Aa-fed mice revealed deficiencies in levels of costimulatory molecules (CD40 and CD80) as well as a poor ability to stimulate Th1 responses *in vitro*. Levels of IL-12 production by stimulated macrophages were lower, suggesting that macrophages from adult Aa-fed mice had features of functionally immature cells. Thus, stimulation by food proteins contributes to APC maturation and to the regulation of an adult type of Th1 immunity to infection. It is tempting to speculate that a similar immature profile of APCs might be present in immature immune states such as in PM.

Likewise, we verified that the susceptibility of Aa-fed mice to the induction of nasal tolerance to ovalbumin (Ova) in a murine model of Ova-induced airway inflammation is also affected (Mucida et al., 2004). Nasal administration of antigens is able to induce specific tolerance and to suppress inflammatory responses to antigens such as eosinophilic airway inflammation in mice fed either a normal chow or a control diet. Aa-fed mice had only a partial suppression of specific IgE production and airway inflammation by the nasal administration of Ova. The immunological immaturity of immune system in these animals seems to be related not just to a deficiency to respond to infectious agents but also to a defective ability to generate immunomodulatory mechanisms.

A curious finding on the food protein effect in the immune system is that although dramatic when their intake is withdrawn at weaning, the immune-stimulatory effect of food proteins can be readily recovered in adulthood. When Aa-fed adult mice are fed a diet containing at least 5% casein for 72 h, they develop normal levels of serum IgG and IgA (Amaral et al., 2006). This indicates that the defect observed in immunological maturation is related to the low antigenic stimulation rather than stimulation at a special period of life. Interestingly, some alterations found in protein-deprived mice are also similar to the ones reported in adult germ-free mice (Jiang et al., 2004) indicating that both food proteins and microbiota antigens have stimulatory effects during the development of the immune system. In Table 2, we compare the immunological changes between whole-protein-deprived diet (Aa diet) and germ-free mice. Although a general effect can be detected for both types of luminal antigens, there are also distinctive immunological effects of food versus microbiota antigens. As expected, immunological alterations in antigen-free mice (germ-free mice reared in an elemental diet) are much more severe (Hooijkaas et al., 1984) suggesting that the formation of the gut lymphoid tissue as well as the establishment of an adult immunological profile are a net result of stimulation by intestinal microbiota and dietary proteins. Of course, food components other than proteins may also play a relevant role in the process.

**Table 2:** **Comparative immunological effects in the absence of natural antigen stimulation (diet protein versus microbiota)**.

Models	Protein-free diet mice	Germ-free mice
Small intestine (villi)	Lost the typical pleated appearance (Menezes et al., 2003)	Thin and more pointed at the tip (Thompson and Trexler, 1971)
Large intestine	No differences (Menezes et al., 2003)	Megacaecum (Thompson and Trexler, 1971)
Peyer patches (PP)	Smaller (Menezes et al., 2003)	Smaller (Thompson and Trexler, 1971)
Lamina propria (LP)	↓Number of lamina propria cells (Menezes et al., 2003)	↓Number of lamina propria cells (Thompson and Trexler, 1971)
Intraepithelial lymphocytes (IEL)	↓Number	↓Number
	↓%TCR αβ	Cytotoxicity compromised (Umesaki et al., 1993; Imaoka et al., 1996)
	↑%TCR γδ (Menezes et al., 2003)	
Immunoglobulins	↓sIgA	↓sIgA
	↓Ig, IgG, and IgA (Menezes et al., 2003)	↓Ig, IgG, and IgA (Nielsen and Friis, 1980)
Infection susceptibility	↑Susceptible to *Leishmania major* (Amaral et al., 2010)	↑Susceptible to *Leishmania major* and others (Oliveira et al., 2005)

*↑, increased; ↓, depressed; secretory IgA (sIgA)*.

Many cellular sensors of bacterial products, amino acids, salt concentration, and plant components have been described as important stress detectors by which multicellular organisms sense and develop strategies to adapt to their environmental challenges (Puga et al., 2009; Jewell and Guan, 2013; Lang and Shumilina, 2013). Specific nutrient receptors expressed by enterocytes, as discussed later in this review, can be also included in this category. Interestingly, several of these environment sensors participate in the development of the lymphoid tissue and in the differentiation of subsets of lymphocytes. Aminoacid signaling through the highly conserved serine-threonine kinase mammalian target of rapamycin (mTOR) has well known consequences on proliferation of immune cells and rapamycin is widely used as an immunossupressor (Jewell and Guan, 2013). Interestingly, this signaling molecule is also connected to other molecular sensors. mTOR is essential for the phosphorylation of the serum- and glucocorticoid-induced protein kinase 1 (SGK1), a kinase that plays a fundamental role in sodium (Na^+^) and potassium transport processes in epithelia (Lu et al., 2010). Two groups have shown that SGK1 is involved in the differentiation of Th17 lymphocytes and administration of a high-salt diet was able to worsen experimental autoimmune encephalomyelitis (EAE) in susceptible mice by the expansion of pathogenic Th17 cells (Kleinewietfeld et al., 2013; Wu et al., 2013). The specific effect of SGK1 signaling in the maintenance of the large population of intestinal Th17 cells has not been addressed yet but it is plausible that dietary salt concentration would have a direct effect on it. Molecular sensing of non-nutritional phytochemicals by the arylhydrocarbon receptor (AhR) is also involved in the maintenance of IELs (Li et al., 2011), and innate lymphoid cells (ILC) (Kiss et al., 2011) in the intestine. AhR is required for the postnatal expansion of intestinal RORγt(+) ILC and the formation of intestinal lymphoid follicles (Kiss et al., 2011). AhR deficiency or the lack of AhR ligands compromises the maintenance of IELs and the control of the microbial load and composition, resulting in increased immune activation and increased vulnerability to epithelial damage (Li et al., 2011). AhR-deficient mice have poorly developed gut follicles and were highly susceptible to intestinal infection with *Citrobacter rodentium* (Kiss et al., 2011). AhR activity within RORγt(+) ILC could be induced by components contained in vegetables of the family Brassicaceae.

## Lipids as Signaling Molecules

The finding that dietary proteins are recognized by MHC by APCs in the gut mucosa with important immunological consequences raises questions about the function of other macronutrients on the activity of the immune system. In fact, recent data show that some lipids have also a relevant role in keeping mucosal homeostasis.

Lipids and glycolipids can be presented by CD1 molecules, a distinct set of antigen-presenting molecules that are evolutionarily related to the classical MHC class I and class II. Unlike the classical MHC products that bind peptides, CD1 binds fatty-acid tails of glycolipids that fit into two hydrophobic pockets of its binding groove (Jayawardena-Wolf and Bendelac, 2001). Using CD1d deficient mice, Hong et al. (1999) demonstrated that CD1d expression is required for the development and action of NKT cells. Invariant NK T cells express a canonical αβTCR with an invariant Vα14-Jα18 rearrangement and they are an important innate-like lymphocyte population programed for CD1d-restricted recognition of glycolipids. Therefore, it is not surprising that NKT cell function can be modulated according to the type of the fatty acid they bind (Barone et al., 1989). However, our understanding of the binding abilities and functions of NKT cells have been constructed mostly on experiments studying immunity against bacterial lipid compositions (Girardi et al., 2011). The effect of dietary lipids, including essential fatty acids, on CD1d presentation and stimulation of T lymphocytes still needs better investigation. This would help to clarify the specific role of lipid stimulation of the population of NKT cells that exist in the liver and in the gut lamina propria. It has been reported that gut NKT cells are required for the survival and expansion of superficial lamina propria B cells. Because these B cells (and invariant NK T cells) are fully preserved in germfree mice, the antigenic drive for this interaction would seem to involve food or endogenous glycolipids, rather than enteric commensal microbial products (Velazquez et al., 2008).

Recently, it has been demonstrated that γδ T cells, another population of innate-like T lymphocytes, have the ability to recognize glycolipids bound by CD1 molecules. Russano et al. (2007) demonstrated that a substantial percentage of TCRγδ+ T cells (60%) isolated from human duodenal mucosa recognize exogenous phospholipids in a CD1-restricted fashion. A Th1-like cytolytic and functional activity along with the ability to secrete regulatory cytokines was observed in most of the isolated phospholipid-specific γδ T cell clones. γδ T cells are abundant among the IELs in the human intestine and they are believed to participate in cytotoxic as well as regulatory functions in the gut mucosa. This adaptive response could contribute to mucosal homeostasis, but could also favor the emergence of inflammatory or allergic intestinal diseases.

The action of lipids as unusual antigens is not the only way that this macronutrient can influence immune responses. After being metabolized, fatty acids are incorporated into membranes of cells including immune cells. The composition of lipids in this cellular structure will affect membrane fluidity and lipid raft formation, influence cell signaling and antigen presentation. Several mechanisms are involved in the role played by membrane lipids in cellular activation including modulation of calcium release after immunological synapse, modification of protein position on lipid rafts, alteration of phosphorylation of specifics enzymes involved in cell signaling as well as changes in MHC conformation, co-stimulation, and adhesion molecule stability (Shaikh and Edidin, 2006). Williams et al. (2012) showed that different fatty acids may have distinct abilities to be incorporated into membranes which will in turn determine their bioactivity. Indeed, others have shown that fish oil increases raft size and membrane organization resulting in functional changes on immune cells (Rockett et al., 2012).

Recent reports have shown that nutrient receptors (such as T1R1/T1R3 heterodimer, Ca^+^ sensing receptor and GPR93 that bind for amino acids and proteins, GPR40, GPR41, GPR43, and GPR120 for fatty acids, T1R2/T1R3 heterodimer for monosaccharides) are expressed in the apical face of the intestinal epithelial cells and sense nutrients in the lumen. They transduce signals for the regulation of nutrient transporter expression in the apical surface of these cells. Moreover, signaling by some of these receptors [such as receptors for short chain fatty acids (SCFAs)] has important anti-inflammatory properties. The SCFAs are the main metabolic products of anaerobic bacteria fermentation in the intestine. These fatty acids have been recognized as potential mediators involved in the effects of gut microbiota on intestinal immune function. SCFAs act on leukocytes and endothelial cells through the activation of GPCRs (GPR41 and GPR43) and inhibition of histone deacetylase (HDAC). SCFAs regulate several leukocyte functions including production of cytokines (TNF-α, IL-2, IL-6, and IL-10), eicosanoids, and chemokines (e.g., MCP-1 and CINC-2). SCFAs can regulate local and systemic inflammatory processes by signaling through GPRs (Maslowski et al., 2009; Vinolo et al., 2011).

Lipid receptors expressed in the population of conventional NK cells may also participate in the signaling of dietary fatty acids. Animal studies revealed that diets containing fish oil or olive oil induce greater inhibition of NK cell activity when compared with diets rich in either saturated fatty acids or n-6 polyunsaturated fatty acids (PUFA) (De Pablo et al., 1998).

In recent years, nutrigenomic studies have received great attention and showed that gene expression can be modulated by dietary fatty acids. In this scenario, the nuclear transcriptional factor peroxisome proliferator activated receptors (PPAR), a member of the steroid nuclear receptor superfamily that binds PUFA has been extensively investigated. Recent studies have shown that the agonist of PPAR may lead to decreased expression of insulin-resistance-inducing adipokines and an anti-inflammatory profile of immune cells such as macrophages by blocking the induction of several pro-inflammatory cytokines such as IL-1α, IL-1β, and IL-6 (Yu et al., 2002; Choi et al., 2010). Expression of PPAR in mouse colonic and small intestinal mucosa is extremely high (Mansen et al., 1996) and this may explain a body of work showing the role of PPAR in the modulation of experimental models of colitis (Katayama et al., 2003; Adachi et al., 2006; Dubuquoy et al., 2006; Celinski et al., 2011). However, most of these studies have used pharmacological approaches, usually applying various synthetic PPAR agonists.

Our group has used a recently discovered dietary fatty acid named conjugated linoleic acid (CLA) to prevent murine models of colitis with great success. The CLA is a mixture of positional and geometric isomers of octadecadienoic acid with conjugated double bonds naturally found in dairy products (Pariza et al., 2001). CLA is a PPAR agonist and has anticarcinogenic, antiatherogenic, antidiabetic, and anti-inflammatory effects (Yu et al., 2002). Previous studies from our laboratory showed that CLA ameliorated intestinal tissue damage in IL-10-deficient mice and colitis induced by dextran sodium sulfate (DSS). CLA-fed mice had lower weight loss during disease development and maintained a gut cytokine profile resembling that of naïve mice. The inhibitory effects of CLA in colitis of IL-10-deficient mice were also associated with expansion of CD4^+^CD25^+^Foxp3^+^ regulatory T cells (Tregs). These data suggest that CLA signaling may contribute to reinforce immunoregulatory mechanisms generated in the gut mucosa (Moreira et al., 2012).

Dietary fat may also have an antiviral therapeutic effect. Recently, Liu and coworkers demonstrated an important link between cholesterol and viral infection. Cholesterol-25-hydroxylase (CH25H) is an interferon-stimulated gene that encodes an enzyme that catalyzes oxidation of cholesterol to a soluble antiviral factor 25-hydroxycholesterol (25HC). Administration of 25HC in humanized mice was able to suppress HIV activity including replication and HIV-induced T cell depletion (Liu et al., 2013).

It is noteworthy that dietary lipids or free fatty acids not only affect the immune system directly through gene modulation, incorporation in cell membrane or lipid presenting cells, but also interfere with the production of other substances such as eicosanoids and anti-oxidants. Fatty acids influence eicosanoid production that act on the immune cells modulating cytotoxicity (Kelley et al., 1999), lymphocyte proliferation (Shapiro et al., 1993; Jeffery et al., 1996; Peterson et al., 1999), and cytokine production (Rappaport and Dodge, 1982; Rola-Pleszczynski and Stankova, 1992).

Unsuspected links between immunity and metabolism have also been found recently by Matzinger’s group. They found that, in the absence of B cells and IgA, but in the presence of the microbiota, the intestinal epithelium launches its own protective mechanisms, upregulating interferon-inducible immune response pathways and simultaneously repressing Gata4-related metabolic functions. This shift in intestinal function leads to lipid malabsorption and decreased deposition of body fat (Shulzhenko et al., 2011).

## Vitamins in Immune Regulation

There are many other components in diet, beyond macronutrients, that affect the activity of immune cells. It has been extensively shown that anti-oxidant vitamins and trace elements (vitamins C, E, selenium, copper, and zinc) counteract potential damage caused by reactive oxygen species to cellular tissues, modulate immune cell function through regulation of redox-sensitive transcription factors, and affect production of cytokines and prostaglandins (Maggini et al., 2007).

Vitamins are essential nutrients needed in low amounts for the normal functioning of the body. Many have direct effects in the immune system. Vitamin E and C are anti-oxidants but also prevent the development of aortic lesions by inhibiting the expression of adhesion molecules and by modulating macrophage and monocyte function (Koga et al., 2004). Some immune cells are capable of metabolizing vitamin D to its active form 1,25-dihydroxyvitamin D3 (1,25(OH)_2_D3), and that is why this vitamin is easily found in lymphoid sites at high concentrations. Vitamin D3 inhibits T cell proliferation, reduces the expression of IL-2, IL-6, IL-23, IFN-γ, and upregulates IL-4 expression. IL-10 production by T cells is also increased in the presence of vitamin D (Mora and von Andrian, 2008). Activation of CD4^+^ T cells results in a fivefold increase in its vitamin D receptor expression, enabling regulation of at least 102 identified genes responsive to 1,25(OH)_2_ vitamin D3. Decrease in IL-6 secretion leads to inhibition of Th17 responses at several levels, including the ability of dendritic cells (DCs) to support priming of Th17 cells and the ability of Th17 cells to produce IL-17. In addition to effects on CD4^+^ T cells, vitamin D facilitates the induction of Foxp3^+^CD25^+^CD4^+^ Tregs and there is a positive correlation between serum vitamin D levels and the ability of Tregs to suppress T cell proliferation. The major effect of this vitamin in the immune system is the inhibition of DC maturation with inhibition of activation molecules such as MHC class II, CD40, CD80, and CD86 and up-regulation of inhibitory molecules (ILT3) (Kamen and Tangpricha, 2010).

Vitamin A is also known as retinol. Apart from its immunomodulatory ability, retinol has a significant anti-oxidant property and it is imperative to the good functioning of the visual system, for growth and development of the whole body, for the maintenance of epithelial cell integrity and for reproduction (Reifen, 2002). Although vitamin A is easily found in many types of food, its deficiency is a world health problem, and the recommended daily consumption for this nutrient is 900–700 micrograms, depending on gender for adults, 770 micrograms for pregnant and 1300 micrograms for lactating women (Otten et al., 2006). Usually vitamin A is ingested as retinol, or in its pro-form, as carotenoid. The most active metabolite of this compound is retinoic acid (RA), which binds to specific nuclear receptors in many cell types (Iwata et al., 2004). RA biosynthesis occurs locally where it is required. The main pathway of RA biosynthesis is dependent on two steps. The first step, from retinol to retinal, is catalyzed by a subfamily of alcohol dehydrogenases (ADH) commonly expressed in most cells (including immune cells). The second step is an irreversible conversion of retinal to RA that is catalyzed by retinal dehydrogenases (RALDH), which are expressed in specific cell types (Iwata et al., 2004). Surprisingly, it was found that some DC populations are capable of converting retinal in RA, besides the capacity to convert retinol in retinal (Iwata et al., 2004). Only gut DCs (from lamina propria, mesenteric lymph nodes, and PP) have the enzyme RALDH to catalyze the second step of the generation of RA (Iwata et al., 2004). There are also specific subsets of lamina propria macrophages that are capable of converting retinal in RA (Manicassamy and Pulendran, 2009). RA has the ability to induce expression of integrin α4β7 and chemokine receptor CCR9, imprinting CD4^+^ and CD8^+^ T lymphocytes with gut-homing specificity (Iwata et al., 2004);(Johansson-Lindbom et al., 2005). It has been also shown that mucosal CD103^+^ DCs are capable of converting Foxp3^-^CD4^+^ naïve T cells into Foxp3^+^ CD4^+^ T cells in the presence of TGF-β (Coombes et al., 2007; Mucida et al., 2007). This conversion occurs by histone acetylation at the *FoxP3* gene promoter and subsequent expression of the FoxP3 protein by CD4^+^ T cells (Kang et al., 2007). Induction of “retinoid-induced FoxP3^+^ T cells” is mediated by the nuclear RA receptor alpha (RARα) and involves T cell activation driven by mucosal DCs and co-stimulation through CD28 (Kang et al., 2007). However, a word of caution on this topic is necessary. Vitamin A effects could vary, depending on the surrounding. DePaolo et al. (2011) showed that, in the presence of the pro-inflammatory cytokine IL-15, usually abundant in the inflamed intestine, RA induces an increase in the production of IL-12p70 and IL-23, which leads to suppression of the differentiation of Tregs and in the increase in Th1 responses with a high IFN-γ production. It seems therefore that RA action depends on the presence and activity of other cytokines.

Many studies have been published on the various effects of RA in the immune system. Some of them have tested feeding vitamin A as dietary supplement (Garcia et al., 2003; Felipe et al., 2005; Kheirvari et al., 2008) instead of intraperitoneal injection of RA (Bai et al., 2010) or intragastrical administration of retinol by gavage (Reifen, 2002). We have examined the effect of a vitamin A-supplemented diet in the model of colitis induced by DSS. Animals fed this supplemented diet showed a milder loss of the body weight than the control group (not supplemented) during the induction of colitis. There was also an increase in the number of CD4^+^FoxP3^+^ Tregs in spleen and mesenteric lymph nodes as well as preserved frequencies of CD103+ DCs in mesenteric lymph nodes whereas non-supplemented animals showed a decrease in these cells (Medeiros et al., 2011). Therefore, our results confirm that vitamin A may have a beneficial effect in preventing inflammatory situations in which mechanisms that keep mucosal homeostasis and tolerance to luminal antigens are disrupted.

## Oral Tolerance as Incorporation of Molecular Novelty through the Gut

There are two major consequences that follow the contact with antigens by the gut lymphoid tissue: production of secretory IgA and induction of oral tolerance. Secretory IgA is a non-inflammatory subclass of Ig that is present in all mucosal secretions providing a mechanism of clearance of pathogenic microorganisms without the inconvenience of inflammatory responses (Macpherson et al., 2008). Oral tolerance is a phenomenon known in medical literature since 1909 when Anton Besredka showed that guinea pigs fed milk-containing chow could not be immunized against milk proteins (Faria and Weiner, 2005). Since this first description until very recently, oral tolerance has been marginally quoted. In the 70’s when suppressor T cells were proposed to explain the inhibitory effect generated by feeding antigens, oral tolerance gained some systematical and important studies (Richman et al., 1978; Faria and Weiner, 2005). With the recent revival of Tregs and their important role in maintaining central as well as peripheral tolerance, the suppression induced by oral administration of antigens came back into the scene. Indeed, oral tolerance has been now extensively described in animal models of inflammatory diseases (Faria and Weiner, 2006) and also in humans (Mestecky et al., 1996). Oral tolerance probably accounts for the robust balance that maintains homeostasis of intestinal mucosa in spite of its highly activated lymphoid tissue (Faria and Weiner, 2005). We are all tolerant to the food proteins we ingest and also to our own microbiota. This has been documented in mice and humans (Mestecky et al., 1996; Andrade et al., 2006; Round et al., 2010).

It has been suggested that oral tolerance to food antigens is able to induce systemic suppression but tolerance to microbiota is restricted to local anti-inflammatory responses associated with secretory IgA production (Macpherson and Uhr, 2004; Pabst and Mowat, 2012). This is an interesting proposition for a number of reasons: (a) the uptake of bacterial products and food proteins seem to occur by distinct mechanisms (Pabst and Mowat, 2012); (b) differently from luminal bacteria, food proteins are known to enter the circulation continuously and reach systemic organs (Husby et al., 1985); (c) most of the lymphocytes in the gut are located in the small intestine where food proteins are mostly absorbed (Mowat, 2003); (d) it has been shown that bacteria do not cause disease if they remain within the intestinal lumen, but they contain abundant immunostimulatory molecules that trigger immunopathology if they are intravenously injected (Macpherson et al., 2005); (e) there are similarities but also differences between germ-free mice and mice reared without intact dietary proteins (Table 2). Therefore, although gut microbiota has a robust effect in maintaining the intestinal homeostasis (Ivanov and Littman, 2011), food proteins would have both local and systemic effect as modulators of the immunological activity through oral tolerance induction.

The mechanisms involved in oral tolerance seem to be similar to the ones triggered during central tolerance in the thymus, i.e., induction of deletion of specific T cells and induction of Tregs (Faria and Weiner, 2005; Weiner et al., 2011). The critical role of Tregs in oral tolerance has been shown by classical transfer experiments (Richman et al., 1978), by the fact that depletion of antigen-specific Tregs prevents oral tolerance induction (Hadis et al., 2011), and by recent findings demonstrating the need of adaptive, but not natural, Foxp3^+^ Tregs (Mucida et al., 2005; de Lafaille et al., 2008). Some different subsets of Tregs have been implicated in oral tolerance induction: IL-10-producing Tr1 cells, TGF-producing Th3 cells, and CD4^+^CD25^+^Foxp3^+^ induced Tregs (Pabst and Mowat, 2012). Indeed, it has been shown that CD4^+^ T cells can be converted into CD25^+^ activated Foxp3^+^ regulatory cells in the intestinal mucosa by the action of CD103^+^ specialized DCs that secrete RA as discussed earlier in this review (Coombes et al., 2007; Mucida et al., 2007). Other types of Tregs are also induced by the contact with intestinal antigens. One recently described subset of gut-induced Treg are the CD4^+^ T cells characterized by their surface expression of the latency-associated peptide (LAP), which is the N-terminal propeptide of the TGF-β precursor (Nakamura et al., 2004). The intestinal mucosa is a privileged site for the generation of Tregs expressing LAP. CD4^+^CD25^+^LAP^+^ and CD4^+^CD25^−^LAP^+^ Tregs promote their suppressive functions mainly in a TGF-β-dependent fashion (Oida et al., 2003; Nakamura et al., 2004; Chen et al., 2008). Besides TGF-β, other inhibitory cytokines such as IL-10 and IL-35 have been reported to participate in the regulatory network of the gut (Kuhn et al., 1993; Vignali, 2008). Indeed, genetic deficiency of IL-10 is associated with disruption in gut homeostasis and severe colitis in mice (Kuhn et al., 1993). Susceptibility to oral tolerance is reduced in IL-10-deficient mice, and it can only be induced by an optimized regimen of oral administration such as continuous feeding (Gomes-Santos et al., 2012).

Among the feeding protocols already tested in oral tolerance experiments, continuous feeding of the antigen has been shown to be the most efficient one. This regimen refers to the procedure of diluting the antigen in the drinking water that will be consumed during the day as opposed to the intragastric administration of an equivalent dose of antigen *in bolus*. Continuous feeding of specific antigens is able to prevent more efficiently the development of myelin basic protein (MBP)-induced EAE and to render tolerant senescent mice that are usually refractory to oral tolerance induced by gavage. Moreover, oral tolerance induced by continuous feeding of antigen lasts longer and, in mice, does not require any type of reinforcement during their lifetime (Faria et al., 1998, 2003). As mentioned before, this regimen of oral administration is able to induce tolerance also in IL-10-deficient mice that is resistant to tolerance induction by gavage (Gomes-Santos et al., 2012). It is interesting to note that continuous feeding is a protocol that resembles the natural feeding process. In concert with this idea, elegant studies by Verhasselt et al. (2008) have shown that exposure of suckling mice to an airborne antigen (Ova) transferred from Ova-fed mothers through breast milk lead to induction of oral tolerance to Ova-induced experimental asthma in the progeny. In addition, breastfeeding the antigen was also able to induce tolerance even when the mothers were already allergic. Breast milk from antigen-exposed sensitized mothers contained antigen-IgG immune complexes that were transferred to the newborn through the neonatal Fc receptor resulting in the induction of antigen-specific FoxP3^+^CD25^+^ Tregs (Mosconi et al., 2010).

As an approach to prevent inflammatory pathological conditions, oral tolerance has been proved successful in many animal models of autoimmune and other inflammatory diseases (Faria and Weiner, 2006). Our group described recently a recombinant hsp65-producing *Lactococcus lactis* that delivers antigen in the intestinal lumen in a continuous feeding fashion. This novel strategy for oral tolerance induction to an inflammation-related antigen inhibited the development of autoimmune experimental encephalomyelitis in mice (Rezende et al., 2013).

From the immunological and biochemical point of view, nutrition represents a daily incorporation of molecular novelty into the body, and it can be considered as a way of continuous self construction. In coordination with that, the GALT has developed thymus-like mechanisms that are able to treat the antigens coming from the diet and from the autochthonous microbiota as if they were self antigens.

Many cell types and mediators seem to participate in this ability of gut mucosa to incorporate new antigens by inducing tolerance. There is a long list of modulatory molecules that are produced locally, but IL-10 and TGF-β are critical cytokines for controlling gut homeostasis. IL-10 is secreted by macrophages, T cells, and B cells, whereas TGF-β 1 is produced by T cells and by many non-lymphoid/myeloid cells, especially epithelial cells. The small and large intestine contain the largest reservoir of macrophages in the body. These cells express F4/80, CD11b, low levels of most innate receptors (CD14, FcγR, TLR1-5, TREM-1), and low-to-modest levels of MHC II. Intestinal macrophages produce IL-10 constitutively and following stimulation with bacteria and are non-inflammatory cells that retain the capacity to phagocytose and kill invading microorganisms (Kelsall et al., 2011). Intestinal DCs are also an important piece in conditioning the gut mucosal milieu since they are critically involved in antigen presentation and induction of T cells with regulatory properties. DCs from Peyer’s patches (PP) and mesenteric lymph nodes (MLN) from fed mice are able to produce IL-10 and TGF-β as well as to drive the differentiation of T cells producing IL-4 and IL-10 (Iwasaki and Kelsall, 1999). CD11b^+^ DCs from PP are unique in their ability to produce IL-10 and to induce the differentiation of these T cell subsets (Iwasaki and Kelsall, 2001). Lamina propria DCs also express mRNA for IL-10 and IFN-γ, but not IL-12, following feeding (Chirdo et al., 2005). Furthermore, DCs from lamina propria and MLN were capable of driving *de novo* induction of CD4^+^Foxp3^+^ Tregs. Some CD4^+^CD25^+^Foxp3^+^ Tregs in PP develop into follicular T helper cells (Tfh) able to help the differentiation of IgA-producing B cells (Tsuji et al., 2009). In addition, half of the B cells that produce IgA in the gut are composed of B1 cells (Kroese et al., 1989; Sutherland and Fagarasan, 2012). This distinct subset of B cells produces most of the natural serum IgM and much of the gut secretory IgA. These cells also produce large amounts of IL-10 (O’Garra and Howard, 1992). The development of B1 cells depends on the nuclear factor of activated T cells c1 (NFATc1). Fagarasan’s group found recently that RA induces the expression of NFATc1, and leads to the increase of the B1 cells (Maruya et al., 2011). Vitamin A-deficient diets result in reduction of NFATc1 expression in B1 cells and in an almost complete loss of the B1 cell compartment. We have already discussed that the unique ability of gut DCs to induce differentiation of CD4^+^CD25^+^Foxp3^+^ Tregs and to drive the expression of gut-homing receptors CCR9 and α4β7 is related to their ability to produce RA. Pieces of evidence exist showing that stromal cells from mesenteric lymph nodes also express high levels of RA-producing enzymes and are able to support the generation of Foxp3^+^ Tregs and to imprint gut-homing properties on T cells (Hammerschmidt et al., 2008; Pabst and Mowat, 2012). It is remarkable that many of the cell types that play a role in maintaining the tolerogenic milieu of the gut are connected by vitamin A and RA action. Like vitamin A, other molecules of the gut tolerogenic circuit are also derived from food components.

**Figure 1:**
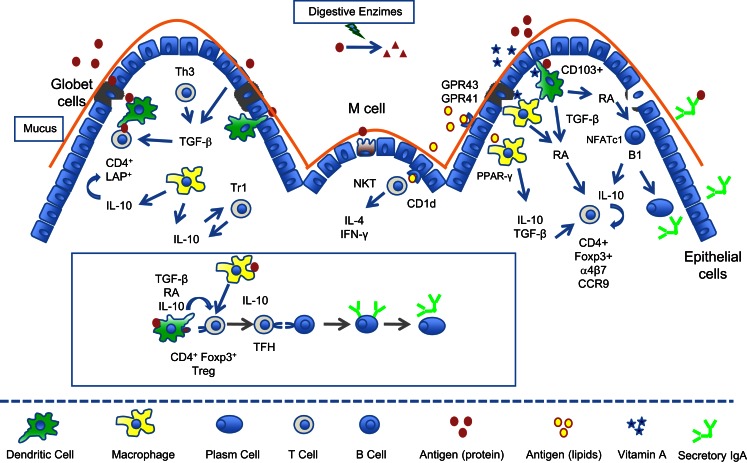
**Immunoregulatory circuits in the gut mucosa triggered by food components**. Food proteins are absorbed either as intact molecules by transcellular transport through specialized microfold cells (M cells) or as peptides by paracellular transport across the epithelial cells. Dendritic cells (DC) and macrophages underlying epithelial cells in Peyer’s patches and in lamina propria take up these antigens and present them inside molecules of class II major histocompatibility complex (MHC II) to naïve CD4^+^ T cells. Most of antigen presentation in the gut mucosa is performed by DCs since resident macrophages have low levels of MHC II. Mucosal CD103^+^ DCs express the enzyme retinal dehydrogenase (RALDH) that convert food-derived vitamin A (retinol) into retinoic acid (RA). Antigen presentation by RA-producing DCs in the presence of TGF-β is able to differentiate naive CD4^+^ T cells into CD4^+^CD25^+^Foxp3^+^ regulatory T cells. Mucosal macrophages have also been shown to produce RA and participate in the generation of Foxp3^+^ Tregs. RA induces expression of α4β7 and CCR9 in T cells imprinting them with gut-homing properties (CD4^+^ and CD8^+^ T cells). RA also induces the expression of NFATc1 in B1 cells, a transcription factor implicated in the development and survival of these cells. TGF-β is an abundant cytokine in the mucosal milieu being secreted by a variety of cells such as epithelial cells, macrophages, DCs, and T cells. This anti-inflammatory cytokine is fundamental for the maintenance of the tolerogenic environment of the gut and it contributes to the differentiation of a number of Tregs such as CD4^+^Foxp3^+^, CD4^+^LAP^+^ T cells as well as TGF-β-secreting Th3 cells. IL-10 is another key cytokine for the maintenance of gut homeostasis and it is produce by several cell types (macrophages, DCs, CD4+ Tr1 cells, and B1 cells). IL-10 modulates macrophage function, helps the differentiation of Th3 cells, and stabilizes the expression of Foxp3 in CD4^+^Foxp3^+^ Tregs. CD4^+^Foxp3^+^ can be converted into follicular helper T cells (TFH) in Peyer’s patches with the help of IL-6 and IL-21. Lipids have also modulatory effects in the gut mucosa by interacting with intracellular PPAR-γ receptors in macrophages. Short chain fatty acids (SCFA) bind to GPR41 or GPR43 metabolic sensors in the epithelial cell surface downmodulating inflammatory responses. Glycolipids may also function as antigens presented by epithelial cells or DCs in CD1d molecules stimulating NKT cells that are able to secrete large amounts of IL-4 and IFN-γ.

Therefore, the GALT uses dietary components (such as lipids and vitamins) as modulatory molecules to maintain gut mucosa at homeostasis and to keep its ability to tolerate innocuous antigens like food proteins (Figure 1) while retaining the capacity to mount a protective response when faced with enteropathogens. Danger molecules in these pathogens probably play a critical role in the differential signaling for inflammatory immune responses (Matzinger, 2007). These molecules may induce the expression of chemokines and inflammatory cytokines resulting in the recruitment of neutrophils, monocytes, and DC precursors from the circulation. The homeostatic balance in the gut can be altered by several factors in the intestine and when disrupted can result in a breach in oral tolerance and consequently in allergic sensitization to food proteins (Chin and Vickery, 2012).

## Food Allergy and Food Allergens

Food allergy is an adverse immunological response triggered by food in susceptible individuals (Wang and Sampson, 2011). The majority of food allergic reactions are caused by IgE-mediated hypersensitivity responses. In this context, the prevalence of IgE-mediated food allergy is 5–8% among newborn and young children under the age of 5 years (Kumar et al., 2012). Genetic factors play a significant role in the predisposition to food allergy. It is demonstrated the occurrence of family history in food allergic patients, high rates of peanut allergy among monozygotic twins, and racial/ethnic determined predispositions (Chin and Vickery, 2012; Kumar et al., 2012). Although the genetic susceptibility clearly participates in the development of food allergy, environmental factors also contribute to the rapid increase in the prevalence of this disease. Lifestyle changes in developed nations may be interfering with the mutualistic relationship between gut bacteria and the host leading to the observed increase in immune mediated disease such as food allergy (Eberl, 2010). Some of these factors that influence the composition and diversity of microbiota are the widespread use of antibiotics, diet composition, elimination of parasitic infections, cesarean birth, and formula feeding (Dethlefsen et al., 2008; De Filippo et al., 2010; Dominguez-Bello et al., 2010; Walk et al., 2010; Yatsunenko et al., 2012). In a genetically susceptible individual, these shifts induce breach of intestinal homeostasis and may predispose to the development of food allergic sensitization.

The mechanism of IgE-mediated food allergy involves two steps: the primary sensitization and the oral challenge. For the first step, APCs especially DCs of lamina propria in intestine capture food allergens (Wang and Sampson, 2011). The allergens are then internalized by DCs and are detected by ubiquitin within the cytosol of these cells. The selective binding of ubiquitin to allergens is the initial signal for specific protein degradation. These ubiquitinized proteins move to proteassomal complex where they are degraded to peptide fragments which are presented by MHC class II (MHC II) to naïve CD4+ T cells (Wang and Maldonado, 2006). As a consequence, the CD4^+^ T cells differentiate into Th2 cells mainly in the presence of adequate amounts of IL-4. The differentiated Th2 cells, in turn, secrete cytokines like IL-4 and IL-13 that induce class switching to IgE on B cells (Kumar et al., 2012). Another pathway involved in IgE-mediated allergic response was described in a model of peanut allergy in mice. In this model, the development of peanut-induced intestinal inflammation is mediated through an IL-13-dependent pathway (Wang et al., 2010). After being secreted, IgE binds to its high affinity receptor FcεRI in mast cells or basophil cells (Kumar et al., 2012).

The second step takes place when the antigen is ingested again and induces the cross-linking of IgE bound to high affinity FcεR1 receptors on mast cells or basophils, leading to degranulation of these cells (Berin and Mayer, 2009). Degranulated mast cells and basophils then secrete several mediators such as prostaglandins, cytokines, leukotrienes, histamine, heparin, platelet-activation factor (PAF), eosinophil chemotactic factor of anaphylaxis, and proteolytic enzymes. Among the mediators of food allergy, various cytokines have been described such as tumor necrosis factor (TNF-α), IL-1, IL-4, IL-5, IL-6, and IL-13. In general, these mediators recruit other leukocytes including eosinophils and Th2 lymphocytes to the site of inflammation which, in turn, contribute to exacerbate the allergic reaction (Minai-Fleminger and Levi-Schaffer, 2009). Recently, IL-33, a newly described member of the IL-1 family has been associated with allergic responses. It was demonstrated that IL-33 induces the production of Th2 cytokines via IL-33 receptor, which is largely expressed on mast cells and Th2 cells (Ohno et al., 2012). In the presence of IgE, IL-33 directly induces degranulation of mast cells playing an important role in anaphylactic shock in mice via an IL-4-dependent pathway (Pushparaj et al., 2009). These mediators are able to induce smooth muscle contraction, increased vascular permeability, leukocyte recruitment, increased mucus secretion. These alterations may also occur very vigorously leading to anaphylaxis and death (Cara et al., 2004; Kumar et al., 2012). Systemic signals, which lead to metabolic changes, are also found in chronic food allergy.

Using an animal food allergy model, we observed several of these allergic signals (Saldanha et al., 2004). It is interesting that, while a protein-free diet increases the susceptibility to *L. major* infection, allergic mice had an increased resistance to infection (Saldanha et al., 2008). Another relevant signal observed was the presence of a significant weight loss 7 days after oral challenge with a concomitant decrease in adipose tissue mass. This decrease has been associated with increased lipolysis and local inflammation. Indeed, in adipose tissue of allergic mice, there was an increase in leukocyte rolling and adhesion in the microvasculature with higher numbers of leukocytes, especially macrophages (F4/80^+^ cells), and increased levels of pro-inflammatory cytokines such as TNF-α, IL-6, and CCL2. Moreover, systemic metabolic changes were noted in allergic mice including decrease in serum concentrations of triglyceride, glucose, total cholesterol, and free fatty acids (Dourado et al., 2011).

Food allergens are generally food proteins from either plant or animal sources. The Food and Agriculture Organization (FAO) and the World Health Organization (WHO) have specified the most allergenic food ingredients, i.e., peanut, soybeans, egg, milk, fish, seafood, wheat, and tree nuts, which are responsible for the majority of food allergic reactions (Kumar et al., 2012). It is known that the food allergens present structural and functional characteristics that play a significant role in determining their ability to trigger allergic responses. Among these characteristics we can mention the small molecular weight, presence of glycosylation residues, lipid binding, and resistance to heat and digestion which ensure the maintenance of the allergenic potential of the protein upon reaching the small intestine (Chin and Vickery, 2012).

In this sense, many food allergens are glycoproteins and it has already been demonstrated that protein glycosylation may contribute to protein stability and enhance immunogenicity (Ilchmann et al., 2010). Another already mentioned common feature of food allergens is lipid binding which is able to protect allergens from degradation and enhance their absorption in the intestinal tract. This is the case of the milk allergen β-lactoglobulin that has been shown to be more stable when bound to lipids (Ruiter and Shreffler, 2012). Moreover, some macromolecular characteristics of protein structure are also known to be important in mucosal immunity. A good example is mammalian milk in which caseins are contained in large micelles and are mainly presented to the immune system via Peyer’s patches potentially promoting antigen sensitization, while whey proteins (β-lactoglobulin and α-lactalbumin) are very soluble and rapidly transported through the intestinal epithelium (Roth-Walter et al., 2008). The immunological action of proteins can also be regulated by medications, such as antacids, which may increase the gastric pH and interfere with the digestive function of the stomach. This physiological interference can lead to the persistence of intact food proteins in the small intestine, which may have an important effect on the development of food allergy. This fact is confirmed by results from a mouse model in which the use of antacids lead to enhanced risk for food allergy development (Pali-Scholl et al., 2010). Furthermore, it has also been reported an increased risk for food specific IgE production in adult patients after antacid treatment (Untersmayr et al., 2005).

As mentioned before, not only proteins, but also other dietary constituents, including vitamin A and lipids have effects in immune homeostasis and there is a growing body of evidence regarding the relationship between diet and the development of allergies. Essential fatty acids have been used for prevention and treatment of allergy symptoms, since the increased prevalence of allergies has been associated with modern dietary style [increased consumption of n-6 PUFA (n-6 PUFA) and decreased n-3 PUFA intake (n-3 PUFA] (Kalyoncu et al., 1999; von Hertzen and Haahtela, 2005). In fact, it has been described that the mucosal damage induced by intestinal hypersensitivity reactions to Ova is regulated by omega-3 fatty-acid enriched diet (Yamashiro et al., 1994). We also have shown that Ova allergic mice had a less severe allergic response when the PUFA omega-3 was increased in the diet. We provided evidence that reduced Ova-specific IgE production by n-3 PUFA supplemented diet lead to a decrease infiltration of eosinophils into the gut mucosa, with mild intestinal inflammatory response in mice. These data altogether suggest that food supplementation with n-3 PUFA may be a promising treatment for food associated allergic disorders in addition to food avoidance and confirm the importance of lipid source for food allergy (de Matos et al., 2012).

Still among the environmental factors, vitamin D deficiency (VDD) is another possible contributor to the increasing prevalence of food allergy. VDD has already been associated with the development of atopic dermatitis and supporting evidence suggests its importance for Treg development as well as mucosal barrier maintenance and repair (Kong et al., 2008; Sidbury et al., 2008; Unger et al., 2009). Indeed, recent data from the National Health and Nutrition Examination Survey revealed that VDD was associated with higher levels of IgE sensitization to peanut in children and adolescents (Sharief et al., 2011). Probably because of their anti-oxidant action, vitamins have also been related to reduced risk of atopy and some symptoms such as wheeze and eczema in infants (Martindale et al., 2005). Altogether we observe that nutrients have a strong impact on the immune system. The question we can ask now is whether the immune system can interfere with diet selection.

## Food Aversion

As mentioned, we observed that allergic condition impacts on the metabolism. Furthermore, it has been demonstrated that allergic sensitization is able to induce an aversive behavior to the allergen. This conclusion is reinforced by studies from our research group showing that mice sensitized to Ova avoid drinking a sweetened egg white solution which is preferred by the control animals (Cara et al., 1994, 1997). This aversive behavior is dependent on IgE production via an IL-4-dependent pathway (Basso et al., 2003; Dourado et al., 2010). Furthermore, it was shown that OVA-sensitized mice avoid entering environments containing traces of OVA and that this behavior is also dependent on IgE and mast cell degranulation (Costa-Pinto et al., 2007). The aversion in a situation of allergic sensitization may be triggered by the memory of a noxious stimulus and is associated with increased levels of anxiety upon exposure to allergen (Costa-Pinto et al., 2005). Recently, the avoidance of the allergen was addressed as a host defense in order to stop the symptoms of allergic reactions (Palm et al., 2012).

A possible mechanism linking allergic response and sensory stimuli is the fact that C-fiber neurons can be activated by histamine and other mediators produced by mast cells upon degranulation. Some possible consequences of this stimulus are itch sensation and other defensive reactions (Jeffry et al., 2011). Thus, the C-fiber stimulation by mast cell products may represent a cross-talk between allergic sensitization and sensory stimuli detected by the olfactory, gustatory, or visual systems (Palm et al., 2012). Regarding the specific aversion to food, a finely developed system of communication between the digestive system and the brain is very plausible. Some studies in mice support this idea. Food allergy induces activation of neurons, evidenced by *c-fos* expression, in the paraventricular nucleus of the hypothalamus (PVN) and central nucleus of amygdala (CeA). These brain areas are related to emotional and affective behavior (Basso et al., 2004). Moreover, activation of CeA and PVN has also been observed in animal models of conditioned taste aversion in which animals avoid the ingestion of saccharin after it had been paired with an intraperitoneal administration of lithium chloride (Yamamoto et al., 1997). One possible mediator of the aversion development, in these cases, is the corticotropin-releasing hormone (CRH), an important peptide in controlling the behavioral, neuroendocrine, and autonomic responses to stress (Holsboer and Ising, 2008; Mirotti et al., 2010).

Therefore, food aversion in the context of food allergy may be viewed as a behavioral adaptive response that results from the interaction of immune and neuroendocrine systems. In this sense, considering the effects of all other dietary components in the immune system, it remains to be investigated whether there is immunological aversion to other diet molecules including lipid and vitamins.

## Concluding Remarks

The gut mucosa is the major interface with the external world and a unique route for incorporation of molecular novelty to the body. It is not a coincidence that this surface houses an equally large lymphoid tissue. Natural antigens, such as food proteins and microbiota components, are strong stimuli for the development of the GALT suggesting that luminal antigens and local immune cells influence each other in a circuit of interactions. Although recent studies have addressed extensively the effects of microbiota antigens in the immune system, only few have shed light to the direct effects of food proteins as natural antigens for the local and systemic immunity. Food proteins seem to trigger non-inflammatory immune responses such as secretory IgA production and oral tolerance with local and systemic effects. It is noteworthy that antigens from gut microbiota seem to induce modulatory immunological mechanisms that are nonetheless restricted to the gut mucosa. We are only beginning to understand the tonic effect of proteins in the physiological activity of the immune system. Moreover, recent studies have demonstrated that other food components, such as lipids, salt, and vitamins, interfere with the development and differentiation of immune cells. Although we still lack a systematic view of these interactions, it is clear that nutrients play a far broader role in the body homeostasis than being building blocks for the metabolism. Exploring these avenues of research will certainly bring along a better knowledge of their biological significance and their potential as therapeutic agents in immune-related diseases such as food allergy.

## Conflict of Interest Statement

The authors declare that the research was conducted in the absence of any commercial or financial relationships that could be construed as a potential conflict of interest.
